# Chromosomal Microarray Diagnostic Yield and Copy Number Variants in a Clinically Well-Characterized Cohort with Nonsyndromic Autism Spectrum Disorder from Southern Brazil

**DOI:** 10.3390/diagnostics16182947

**Published:** 2026-09-11

**Authors:** Willian de Souza Santos, Bonald Cavalcante de Figueiredo, Rosiane Guetter Mello, Sérgio Antoniuk, Gustavo Manoel Schier Dória, Maria Cecília Beltrame Carneiro, Mara L. Cordeiro

**Affiliations:** 1 Faculdades Pequeno Príncipe, Curitiba 80230-020, Paraná, Brazil; willian.santos@aluno.fpp.edu.br (W.d.S.S.); bonald.figueiredo@professor.fpp.edu.br (B.C.d.F.); rosiane.mello@fpp.edu.br (R.G.M.); 2 Instituto de Pesquisa Pelé Pequeno Príncipe, Curitiba 80250-060, Paraná, Brazil; 3 Centro de Neuropediatria do Hospital de Clínicas, Universidade Federal do Paraná (UFPR), Curitiba 80060-270, Paraná, Brazil; antoniuk@uol.com.br (S.A.); gustavodoria@ufpr.br (G.M.S.D.); mariacica@gmail.com (M.C.B.C.); 4 Programa de Pós-Graduação em Saúde da Criança e do Adolescente, Universidade Federal do Paraná (UFPR), Curitiba 80060-270, Paraná, Brazil; 5 Department of Psychiatry and Biobehavioral Sciences, David Geffen School of Medicine at UCLA, University of California, Los Angeles, CA 90095, USA

**Keywords:** autism spectrum disorder, chromosomal microarray analysis, copy number variants, diagnostic yield, nonsyndromic autism, genetic testing

## Abstract

**Background/Objectives:** Chromosomal microarray analysis (CMA) is widely used in the genetic evaluation of autism spectrum disorder (ASD), yet its diagnostic contribution in clinically nonsyndromic ASD, particularly in Brazilian populations, remains insufficiently characterized. This study aimed to determine the diagnostic yield of CMA and characterize clinically relevant copy number variants (CNVs) in a cohort of individuals with clinically nonsyndromic ASD from southern Brazil. **Methods:** In this observational cohort study, 215 individuals with clinically diagnosed ASD underwent high-resolution CMA using the Agilent CGH + SNP Array 180K platform. Individuals with dysmorphic features, known or suspected genetic syndromes, major congenital malformations, epilepsy, or macrocephaly/microcephaly were excluded. CNVs were classified according to American College of Medical Genetics and Genomics guidelines, and the diagnostic yield was compared with that of national and international reference cohorts using Fisher’s exact test. **Results:** Pathogenic or likely pathogenic CNVs were identified in six individuals, corresponding to a diagnostic yield of 2.8% (95% CI, 1.3–6.1%). These included deletions at 2q11.1–q11.2, 2p16.3 (*NRXN1*), 15q11.2 (BP1–BP2), and 3q29, and duplications at 16p11.2 and 6p22.3–p22.2. Although the diagnostic yield was lower than that reported in phenotypically heterogeneous ASD cohorts, it did not differ significantly from yields observed in comparably selected nonsyndromic subgroups. **Conclusions:** CMA identified clinically relevant recurrent CNVs at neurodevelopmental loci even in a clinically nonsyndromic ASD cohort. The comparatively low diagnostic yield highlights the substantial influence of clinical and phenotypic selection on the diagnostic contribution of CMA.

## 1. Introduction

Autism spectrum disorder (ASD) is a neurodevelopmental condition characterized by persistent difficulties in social communication and social interaction, together with restricted and repetitive patterns of behavior, interests, or activities, with symptoms emerging during the early developmental period [1]. The reported prevalence of ASD has increased substantially over recent decades, with recent estimates indicating that approximately 2.3% of 8-year-old children in the United States meet diagnostic criteria for ASD [2]. In Brazil, epidemiological data on ASD were historically limited to a small number of studies conducted in restricted geographic regions; however, the inclusion of a specific question on ASD diagnosis in the 2022 Brazilian Population Census enabled, for the first time, nationally representative estimates. According to preliminary results released by the Brazilian Institute of Geography and Statistics (IBGE), an estimated 2.4 million individuals in Brazil (1.2% of the population aged 2 years or older) reported a diagnosis of ASD, with higher prevalence among males (1.5%) than females (0.9%) and the highest prevalence observed among children aged 5–9 years (2.6%) [3].

The etiology of ASD is complex and multifactorial, with a substantial genetic contribution. Heritability estimates range from approximately 70% to 90% [4,5], and numerous genomic variants have been implicated in ASD susceptibility [6]. Among the genetic alterations associated with ASD are copy number variants (CNVs), defined as submicroscopic deletions or duplications of genomic segments that may alter the dosage of genes involved in neurodevelopment [7,8].

Chromosomal microarray analysis (CMA) has been widely recommended as a first-tier genetic test in the evaluation of individuals with ASD and other neurodevelopmental disorders because of its ability to detect submicroscopic chromosomal imbalances that are not identifiable by conventional karyotyping [9,10]. The diagnostic yield of CMA varies substantially according to the clinical characteristics of the population studied, with higher yields generally reported in individuals with dysmorphic features, congenital anomalies, intellectual disability, epilepsy, or other features suggestive of a syndromic or complex neurodevelopmental presentation [8,11,12].

Despite the increasing use of genomic testing, data on CMA findings in Brazilian individuals with ASD remain limited. A recent large retrospective study from southern Brazil reported a CMA diagnostic yield of approximately 10% among 333 individuals with ASD; however, the sample was derived from a broader referral population that included individuals with neurodevelopmental disorders and congenital anomalies [11]. More broadly, comparisons of CMA diagnostic yield across ASD studies are complicated by substantial phenotypic heterogeneity, as cohorts frequently differ in the inclusion of individuals with dysmorphic features, congenital anomalies, epilepsy, intellectual disability, and other indicators of syndromic or complex neurodevelopmental presentations. Consequently, the diagnostic contribution of CMA specifically in clinically nonsyndromic ASD remains incompletely characterized.

Therefore, the present study was designed to address this gap by evaluating CMA findings in a cohort of individuals with clinically nonsyndromic ASD from southern Brazil. Participants with dysmorphic features, known or suspected genetic syndromes, major congenital malformations, epilepsy, or macrocephaly/microcephaly were excluded. We aimed to determine the diagnostic yield of CMA, characterize the pathogenic and likely pathogenic CNVs identified, examine their associated clinical and psychiatric phenotypes, and compare the observed diagnostic yield with that reported in national and international reference cohorts. By focusing on a clinically nonsyndromic ASD population, this study sought to further clarify how phenotypic selection influences the diagnostic contribution of CMA.

## 2. Materials and Methods

### 2.1. Study Design and Setting

This study was a retrospective secondary analysis of prospectively collected clinical and genetic data from an observational cohort established within the framework of the multicenter project “Intellectual Disability and Autism: Clinical and Genetic Characterization of a Population from Southern Brazil”. The study was conducted at Hospital Pequeno Príncipe, Faculdades Pequeno Príncipe, Instituto de Pesquisa Pelé Pequeno Príncipe and Centro de Neuropediatria do Hospital de Clínicas da Universidade Federal do Paraná (UFPR), in Curitiba, Paraná, Brazil.

### 2.2. Participants

A total of 235 children and adolescents with a clinical diagnosis of ASD were recruited from outpatient clinics in Curitiba and surrounding municipalities in the metropolitan region. Participants of both sexes were eligible for inclusion and were aged 6 to 18 years at enrollment. Before inclusion in the genomic analysis, all participants underwent clinical evaluation by the study neuropediatrician and were classified as having clinically nonsyndromic ASD. Individuals with dysmorphic features, known or suspected genetic syndromes, major congenital malformations, epilepsy, or macrocephaly/microcephaly were excluded prior to CMA testing. Of the 235 participants initially recruited, 20 did not undergo CMA because they did not attend the scheduled sample collection or withdrew from the study, resulting in a final cohort of 215 participants. All participants met DSM-5 criteria for ASD requiring Level 1 support.

### 2.3. Chromosomal Microarray Analysis

Peripheral blood samples were collected in EDTA tubes and centrifuged at 600 g to separate the leukocyte buffy coat, which was stored at −80 °C until DNA extraction. Genomic DNA was extracted using a commercial silica column-based kit (PureLink™ Genomic DNA Mini Kit, Invitrogen (Waltham, MA, USA); Mammalian Cells and Blood Lysate Protocol), according to the manufacturer’s instructions, and stored at −30 °C prior to shipment. DNA concentration was determined by fluorometry (Qubit 3 Fluorometer, Qubit dsDNA BR Assay Kit, Invitrogen) and spectrophotometry (NanoDrop One Microvolume UV-Vis Spectrophotometer, Thermo Fisher, Waltham, MA, USA), with A260/280 and A260/230 absorbance ratios used to assess DNA purity. Samples were shipped as pre-extracted genomic DNA; extraction was performed in-house and was not repeated by the reference laboratory.

Chromosomal microarray analysis (CMA) was performed by a+ Medicina Diagnóstica (Grupo Fleury, São Paulo, Brazil), an ISO 9001-certified [13] laboratory network accredited by PALC (Programa de Acreditação de Laboratórios Clínicos, SBPC/ML) and CAP (College of American Pathologists), using a high-resolution genome-wide platform (Agilent^®^ CGH + SNP Array 180K (Agilent Technologies, Santa Clara, CA, USA); proprietary pipeline_CGH_v.5.1.2). The platform detects copy number variants (CNVs) in selected genes and across genomic segments larger than approximately 200 kb, and, through selected single-nucleotide polymorphism (SNP) probes, identifies uniparental isodisomy and regions of absence of heterozygosity (AOH) spanning more than 10 Mb. Genomic coordinates were based on the GRCh37 (hg19) reference genome. All CNV/AOH calling and classification were performed internally using the laboratory’s proprietary, validated pipeline; probe spacing, internal quality-control thresholds, and the minimum number of consecutive probes required to call a CNV are embedded parameters of this pipeline and are not disclosed in the clinical report format provided to the referring physician.

CNVs were classified according to the American College of Medical Genetics and Genomics (ACMG) and Clinical Genome Resource (ClinGen) technical standards for the interpretation and reporting of constitutional copy-number variants [8], as pathogenic, likely pathogenic, variant of uncertain significance (VUS), likely benign, or benign, based on an integrated evaluation of gene content, dosage sensitivity, overlap with established genomic disorders, evidence from public databases (DECIPHER, DGV, ClinGen, ClinVar, OMIM), and the current literature.

AOH regions exceeding 10 Mb were reported separately from CNV findings and were not classified using the ACMG/ClinGen CNV framework [8], consistent with laboratory practice for this platform. The reported segment represented the largest contiguous AOH region detected in each case; smaller regions below the platform’s individual reporting threshold were incorporated into an overall estimate of genome-wide homozygosity (percentage of total autosomal genome in AOH), as provided by the laboratory. Percentages within a range not considered indicative of excessive homozygosity were not flagged as suggestive of parental consanguinity, in accordance with established thresholds in the literature. Genes within AOH regions were reviewed against disease-gene databases for association with recessive or imprinting-related conditions. Where an AOH finding involved the X chromosome, the laboratory’s copy-number notation (indicating two allelic copies, consistent with 46,XX constitution) confirmed true homozygosity rather than physiological male hemizygosity. Clinically relevant findings were not subjected to orthogonal confirmation (e.g., qPCR or FISH), consistent with standard practice for findings already established and validated by the reference laboratory’s accredited pipeline.

### 2.4. Clinical Assessment and Data Collection

ASD diagnoses were established by participating child psychiatrists following comprehensive clinical assessment and were based on the diagnostic criteria of the Diagnostic and Statistical Manual of Mental Disorders, Fifth Edition (DSM-5; American Psychiatric Association) [1]. Psychiatric comorbidities were assessed by the same psychiatrists using the Schedule for Affective Disorders and Schizophrenia for School-Age Children, Present and Lifetime Version (K-SADS-PL) [14]. Neurological evaluation, including the assessment of dysmorphic features, known or suspected genetic syndromes, epilepsy, and other neurological exclusion criteria, was performed by participating pediatric neurologists following current clinical recommendations for the evaluation of individuals with ASD [15]. Psychologists administered the Portuguese versions of the Autism Diagnostic Interview–Revised (ADI-R) [16] and the Autism Diagnostic Observation Schedule, Second Edition (ADOS-2) [17] for ASD diagnostic characterization, as well as the Wechsler Intelligence Scale for Children, Fourth Edition (WISC-IV) [18] for cognitive assessment. Clinical and behavioral data were collected by the multidisciplinary research team.

### 2.5. Statistical Analysis

Descriptive analyses were performed for all clinical and genetic variables. Categorical variables are presented as absolute frequencies and percentages. The diagnostic yield of CMA was expressed as a proportion with a 95% confidence interval calculated using the Wilson score method. The proportion of pathogenic or likely pathogenic CNVs identified in the present cohort was compared with those reported in national and international reference cohorts, both overall and, where available, within nonsyndromic or least dysmorphic subgroups [11,19,20], using Fisher’s exact test. Psychiatric comorbidities identified using the K-SADS-PL were summarized descriptively for the overall cohort and for participants with pathogenic or likely pathogenic CNVs. All statistical tests were two-sided, and a *p* value < 0.05 was considered statistically significant. Given the exploratory and descriptive nature of the comparisons across six reference cohorts, *p* values were not adjusted for multiple comparisons, and nominal, unadjusted *p* values are reported throughout. Data were organized and tabulated using Microsoft Excel (Microsoft Corporation, Redmond, WA, USA), and confidence intervals and Fisher’s exact tests were calculated using Python 3.12 with the SciPy 1.16 and statsmodels libraries 0.15.

### 2.6. Ethics

The study was approved by the Research Ethics Committee of Faculdades Pequeno Príncipe (CEP/FPP; approval No. 2.493.486; CAAE 70043317.4.0000.5580, approval date 9 February 2018) and by the Brazilian National Research Ethics Commission (CONEP; approval No. 2.450.903, approval date 21 December 2017), in accordance with Brazilian National Health Council Resolution No. 466/2012. Participants were originally enrolled in the multicenter parent project “Intellectual Disability and Autism: Clinical and Genetic Characterization of a Population from Southern Brazil.” Written informed consent was obtained from parents or legal guardians before clinical evaluation and biological sample collection, and assent was obtained from minors aged 7 years or older using age-appropriate assent forms. Participation was voluntary, and parents or legal guardians could withdraw consent at any time without prejudice to the participant’s ongoing clinical care. All data were anonymized before analysis, and participants were identified only by numeric codes. Clinically relevant genetic findings were disclosed to participants and their families, accompanied by individualized genetic counseling.

### 2.7. Use of Generative Artificial Intelligence

During manuscript preparation, Claude Sonnet 5 (high effort mode; Anthropic, San Francisco, CA, USA) was used by the first author solely for English-language editing and refinement and to assist in the preparation of the graphical abstract. The tool was not used for study design, data collection, data generation, statistical analysis, or data interpretation. All AI-assisted output was critically reviewed and edited by the authors, who take full responsibility for the accuracy, integrity, and content of the manuscript.

## 3. Results

### 3.1. Cohort Characteristics

**Figure 1:**
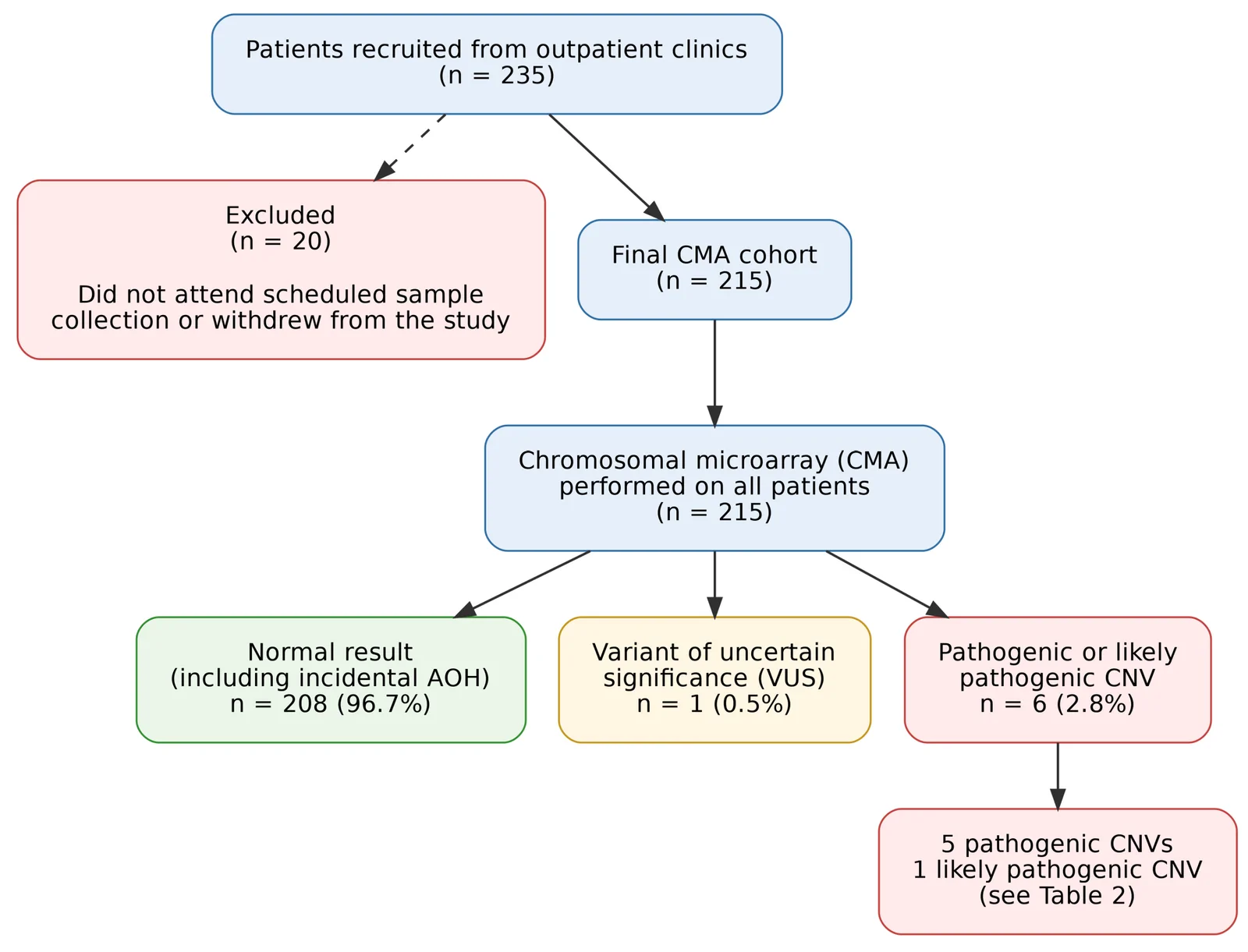
Flow diagram of study participants, from recruitment through chromosomal microarray (CMA) results. The dashed arrow indicates excluded participants, whereas solid arrows indicate the flow of included participants through CMA testing and classification. Box colors visually distinguish exclusion and the different CMA result categories.

Of the 235 patients recruited, 215 underwent CMA and were included in the final analysis (Figure 1). The remaining 20 patients did not undergo testing because they did not attend the scheduled sample collection or withdrew from the study. The cohort consisted predominantly of male patients, consistent with the approximately 4:1 male-to-female ratio reported for ASD in the literature. All participants met diagnostic criteria for ASD requiring Level 1 support. The demographic and diagnostic characteristics of the cohort are summarized in Table 1.

### 3.2. Genomic Findings

**Figure 2:**
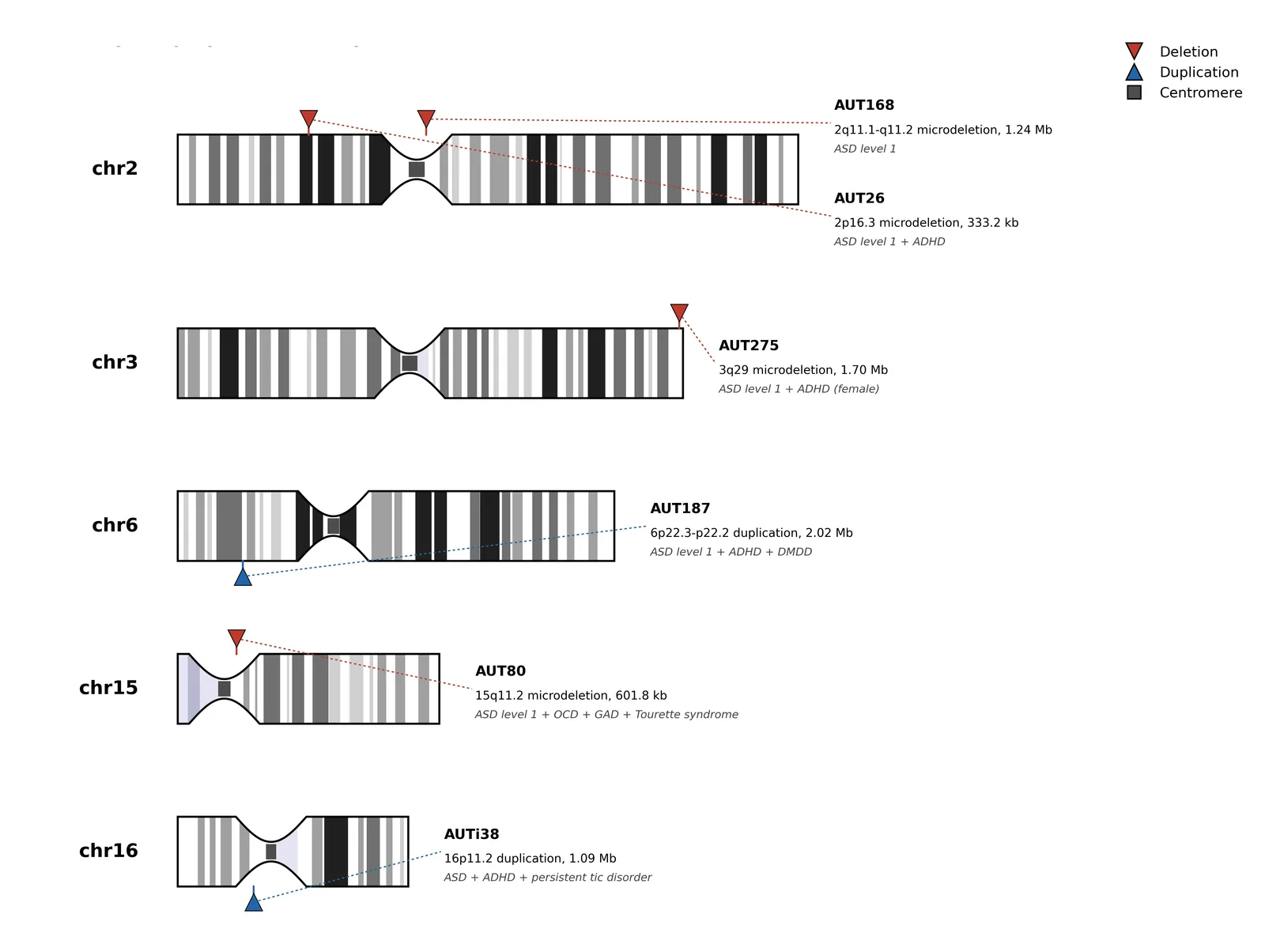
Chromosomal ideogram showing the location of the six pathogenic/likely pathogenic CNVs identified in this cohort. Red markers indicate deletions; blue markers indicate duplications. Chromosome banding is illustrative. Positions are based on GRCh37/hg19 coordinates.

Pathogenic or likely pathogenic CNVs were identified in 6 of the 215 participants, corresponding to a diagnostic yield of 2.8% (95% CI, 1.3–6.1%; Wilson score method). Five participants harbored pathogenic CNVs, whereas one harbored a likely pathogenic CNV. All identified CNVs involved genomic regions previously associated with ASD or other neurodevelopmental disorders. The genomic findings are summarized in Table 2, and the chromosomal locations of the identified CNVs are shown in Figure 2.

In addition, one variant of uncertain significance (VUS), a 1.8 Mb deletion at 4q32.1, was identified and was not included in the diagnostic yield, in accordance with current ACMG classification criteria. Regions of absence of heterozygosity (AOH) larger than 10 Mb were identified in three participants (Xp11.4–p11.21, 15.37 Mb; 22q12.1–q13.31, 20.79 Mb; and 14q13.1–q22.1, 17.75 Mb). None of these regions involved loci associated with recognized imprinting disorders.

### 3.3. Psychiatric Comorbidities in the Overall Cohort

Psychiatric comorbidities were assessed by psychiatrists participating in the study using the K-SADS-PL in 210 of the 215 participants included in the final cohort. ADHD was the most frequent psychiatric comorbidity, identified in 111 participants (52.9%), followed by specific phobia (36.7%), anxiety disorders (19.5%), tic disorders or Tourette syndrome (16.7%), depressive disorders or dysthymia (9.5%), enuresis (7.6%), encopresis (6.2%), and oppositional defiant disorder (3.8%). These findings are summarized in Table 3.

### 3.4. Comparison of CMA Diagnostic Yield with Reference Cohorts

**Figure 3:**
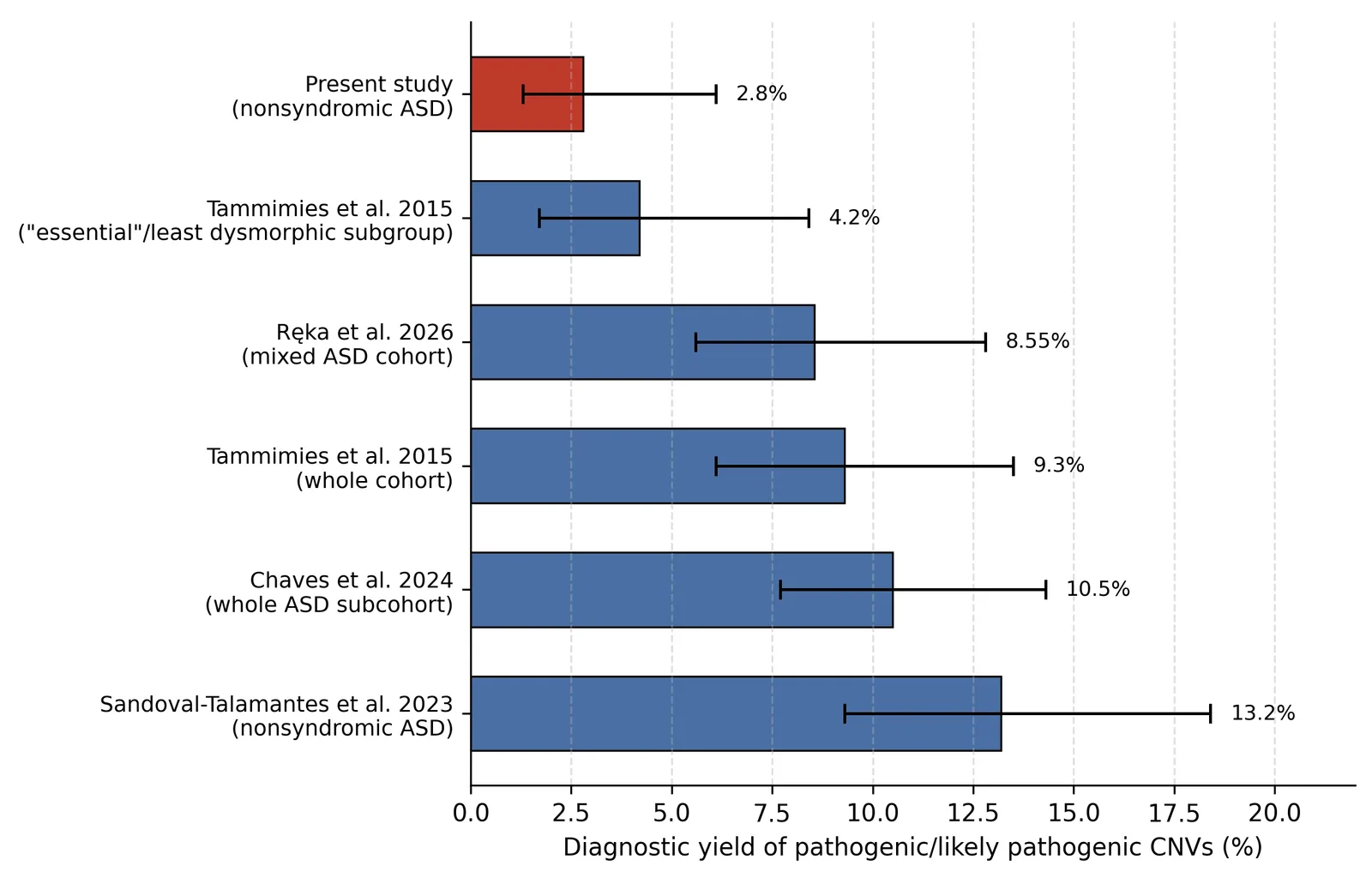
Diagnostic yield of chromosomal microarray in the present study compared with international and national reference cohorts. Error bars represent 95% confidence intervals (Wilson score method) calculated from the counts reported in each original publication [11,19,20,21]. The red bar represents the present study, whereas the blue bars represent the reference cohorts.

The diagnostic yield observed in the present cohort was compared with that reported in national and international reference cohorts. The yield did not differ significantly from that reported in the least dysmorphic (“essential”) ASD subgroup of Tammimies et al. [20] (4.2% vs. 2.8%; Fisher’s exact test, OR 0.66, *p* = 0.57). The diagnostic yield reported for the isolated-ASD subgroup of Chaves et al. [11] was numerically higher than that observed in the present cohort (approximately 7% vs. 2.8%; OR 0.40), although the difference did not reach conventional statistical significance (Fisher’s exact test, *p* = 0.12). In contrast, the diagnostic yield was significantly lower than that reported in the overall, phenotypically heterogeneous cohorts of Tammimies et al. [20] (9.3%; OR 0.28, *p* = 0.004), Sandoval-Talamantes et al. [19] (13.2%; OR 0.19, *p* < 0.001), Chaves et al. [11] (10.5%; OR 0.24, *p* < 0.001), and Ręka et al. [21] (8.55%; OR 0.31, *p* = 0.014). These comparisons are shown in Figure 3. Table 4 summarizes the clinical definitions, selection criteria, sample sizes and diagnostic yields of these reference cohorts.

### 3.5. Familial Occurrence of ASD Within the Cohort

Information on ASD among relatives was collected at enrolment in the original study and was reviewed here for the 215 participants. Twenty-four participants (11.2%) belonged to 12 sibships in which two or more siblings met diagnostic criteria for ASD, were enrolled and underwent CMA. Five further participants had a sibling who was assessed in the study but did not enter the final CMA cohort, either because ASD was not confirmed on standardized assessment or because CMA was not performed, so that 29 participants (13.5%) had at least one sibling assessed. A parent with ASD was recorded for 9 participants (4.2%): 6 mothers and 3 fathers. Taking parents and siblings together, 31 participants (14.4%) had at least one affected first-degree relative, and 60 (27.9%) had at least one affected relative of any degree (Table 5).

Three of the six participants with a pathogenic or likely pathogenic CNV (AUT26, AUT168 and AUT187) had a relative with a reported ASD diagnosis, and none had a parent with ASD. Two of them had a sibling assessed in the study. AUT187, who carries the 6p22.3–p22.2 duplication, has a sibling (AUT188) who also met diagnostic criteria for ASD but whose CMA was normal, so the duplication does not segregate with the phenotype in this family. The sibling of AUT26, who carries the 2p16.3 deletion involving *NRXN1*, had been referred with a previous clinical diagnosis of ASD but did not meet criteria on any of the standardized instruments and did not undergo CMA. No molecular segregation analysis was performed in either family, so these observations rest on clinical phenotype and, in one case, on the sibling’s CMA result.

## 4. Discussion

The present study identified pathogenic or likely pathogenic CNVs in 2.8% of individuals with clinically nonsyndromic ASD. Despite the comparatively low diagnostic yield, CMA identified recurrent and clinically relevant genomic alterations in a carefully selected cohort characterized by the absence of dysmorphic features, recognized or suspected genetic syndromes, major congenital malformations, epilepsy, and macrocephaly or microcephaly. These findings highlight the substantial influence of phenotypic selection on the diagnostic yield of CMA and demonstrate that clinically relevant CNVs can still be identified in individuals with apparently nonsyndromic ASD. Although the diagnostic yield reported for the isolated-ASD subgroup by Chaves et al. was numerically higher than that observed in the present cohort (approximately 7% vs. 2.8%, representing an approximately 2.5-fold difference), this difference did not reach conventional statistical significance (Fisher’s exact test, *p* = 0.12). This comparison should nevertheless be interpreted cautiously given the relatively small number of pathogenic findings and, importantly, differences in the clinical definitions and phenotypic selection criteria used to define the two ASD populations. In particular, the ‘isolated ASD’ subgroup reported by Chaves et al. and the clinically nonsyndromic ASD cohort evaluated in the present study should not be considered directly equivalent.

Beyond the overall diagnostic yield, the specific CNVs identified in this cohort (Table 2) illustrate the heterogeneous genomic architecture underlying ASD. The 2q11.1–q11.2 deletion and the 2p16.3 deletion disrupting *NRXN1* both involve genes with well-established haploinsufficiency evidence; genotype–phenotype studies of *NRXN1* exon-level deletions indicate that disruptions overlapping the α-promoter and initial coding exons, as observed in AUT26, are among those most consistently associated with neurodevelopmental phenotypes, although reduced penetrance and variable expressivity remain characteristic of this locus [22,23]. The 16p11.2 duplication (AUTi38) corresponds to one of the best-replicated recurrent CNVs in ASD and related neuropsychiatric disorders [24], while the 3q29 deletion (AUT275) is notable for conferring one of the highest odds ratios for schizophrenia among known structural variants, in addition to its established association with ASD and intellectual disability [25]. The 6p22.3–p22.2 duplication (AUT187), classified as likely pathogenic largely on the basis of absence from control populations rather than an established triplosensitivity mechanism, illustrates the comparatively weaker evidence base still available for gains relative to losses within current CNV interpretation frameworks.

The 15q11.2 (BP1–BP2) deletion identified in AUT80 merits separate comment. Although classified as pathogenic in the reference laboratory report, this recurrent CNV is widely regarded in the literature as a low-penetrance susceptibility locus rather than a fully penetrant pathogenic variant, with population-based penetrance estimates around 10% or lower and frequent inheritance from unaffected or mildly affected parents [26,27]. Current ClinGen dosage-sensitivity curation of this region reflects population-level, subclinical evidence rather than a discrete haploinsufficient gene mechanism, explicitly distinguishing it from classical high-penetrance microdeletion syndromes. We therefore interpret this finding as a genetic risk allele contributing, alongside the patient’s additional psychiatric comorbidities (Table 2), to a multifactorial neurodevelopmental phenotype, rather than as a standalone causal diagnosis, a distinction with direct relevance for genetic counseling and recurrence-risk estimation. Notably, this classification predates the 2023 publication of the ClinGen Low Penetrance/Risk Allele Working Group framework, which formally established a distinct “risk allele” category for structural variants with this evidentiary profile, separate from traditional pathogenic classification [28]. As the reference laboratory report for this participant was issued in 2022, prior to this framework’s publication, its classification followed the ACMG/ClinGen technical standards in effect at the time [8], which did not yet formally distinguish risk alleles from traditional pathogenic CNVs.

The familial data collected in the original study add context to these findings. A first-degree relative with ASD was recorded for 14.4% of participants and an affected relative of any degree for 27.9%, in keeping with the familial aggregation typical of ASD. None of the six participants carrying a pathogenic or likely pathogenic CNV had a parent with ASD, and in the one family where such a participant had an affected sibling who was also tested, the duplication was present in only a single affected sibling (Section 3.5). A single family cannot support a general conclusion, but the observation fits the view that several of the recurrent CNVs identified here act as susceptibility alleles of incomplete penetrance rather than as fully penetrant monogenic causes. Establishing inheritance would require molecular segregation analysis, which was not performed here.

The comparatively low diagnostic yield observed in our cohort should be interpreted in the context of the substantial phenotypic heterogeneity across published ASD cohorts. As shown by the comparisons presented in Section 3.4, the diagnostic yield in the present study did not differ significantly from that reported in the least dysmorphic (“essential”) ASD subgroup described by Tammimies et al. [20] or from the isolated-ASD subgroup reported by Chaves et al. [11]. In contrast, significantly higher yields were observed in broader cohorts that included individuals with more heterogeneous or complex neurodevelopmental phenotypes [11,19,20,21]. Taken together, these findings suggest that differences in clinical and phenotypic selection account for a substantial proportion of the variability in CMA diagnostic yield reported across ASD studies.

This interpretation is supported by the phenotypic composition of the reference cohorts. In the study by Tammimies et al. [20], the diagnostic yield increased substantially across dysmorphology strata, whereas Chaves et al. [11] reported a higher yield among individuals with syndromic ASD than among those with isolated ASD. Moreover, even cohorts described as nonsyndromic or primary ASD were not necessarily restricted to individuals without additional clinical features associated with an increased likelihood of identifying a genetic etiology. For example, the “essential” ASD subgroup described by Tammimies et al. [20] included individuals with macrocephaly and major congenital anomalies, whereas the cohort reported by Sandoval-Talamantes et al. [19] included individuals with intellectual disability or psychomotor delay and epilepsy. Similarly, a cohort study of genetic testing requests in children with ASD by Garrido-Torres et al. [29] reported comparable heterogeneity in the clinical indications underlying referral for genetic evaluation. By contrast, the present study applied more restrictive clinical selection criteria, excluding individuals with dysmorphic features, recognized or suspected genetic syndromes, major congenital malformations, epilepsy, and macrocephaly or microcephaly. This more stringent phenotypic selection likely contributed to the comparatively lower diagnostic yield observed in our cohort.

The findings also underscore the importance of interpreting CMA diagnostic yield within the specific clinical context in which testing is performed. A lower yield in a clinically homogeneous, nonsyndromic cohort should not necessarily be interpreted as evidence of reduced biological relevance of CNVs in ASD, nor as a population-specific difference. Rather, it may reflect the exclusion of phenotypic features that are themselves associated with a higher probability of identifying pathogenic genomic alterations. The lack of significant differences between the present cohort and comparably selected nonsyndromic subgroups supports this interpretation and emphasizes the need for careful consideration of cohort composition when comparing diagnostic yields across studies.

In this context, the role of CMA in individuals with isolated or clinically nonsyndromic ASD warrants a nuanced interpretation. Although the overall diagnostic yield may be lower than that observed in individuals with syndromic or complex neurodevelopmental presentations, the identification of a pathogenic or likely pathogenic CNV can still have substantial clinical value for the individual patient and family. A molecular diagnosis may inform prognosis, guide surveillance for associated medical or psychiatric manifestations, support recurrence-risk assessment, and facilitate genetic counseling. Thus, the clinical contribution of CMA cannot be fully captured by the diagnostic-yield percentage alone.

At the same time, the comparatively low yield observed in this clinically selected cohort highlights the potential complementary role of sequencing-based approaches. In the cohort reported by Tammimies et al. [20], whole-exome sequencing identified additional molecular diagnoses beyond those detected by CMA, illustrating the contribution of sequence-level variants to the genetic architecture of ASD. Clinical exome or genome sequencing may therefore provide additional diagnostic value in individuals with negative CMA findings, including those with apparently nonsyndromic presentations. Nevertheless, CMA remains particularly suited to the detection of recurrent and submicroscopic genomic imbalances, including the clinically relevant CNVs identified in the present cohort, supporting a complementary or stepwise genomic approach tailored to the clinical phenotype, available resources, and evolving diagnostic guidelines.

### 4.1. Limitations

This study has several limitations. Although the clinical and genetic data were prospectively collected within the framework of the original multicenter study, the present investigation represents a retrospective secondary analysis of these previously collected data. Consequently, the variables available for the current analyses were limited to those collected under the original study protocol. In addition, the deliberate selection of a clinically homogeneous cohort of individuals with nonsyndromic ASD requiring Level 1 support may limit the generalizability of the observed diagnostic yield to broader ASD populations, particularly those including individuals with more complex neurodevelopmental, neurological, or syndromic presentations. However, this phenotypic homogeneity also represents a strength of the study, as it allowed the diagnostic contribution of CMA to be evaluated in a more narrowly defined ASD population.

Comparisons with reference cohorts were based on nominal, unadjusted *p*-values; because several comparisons were performed, the borderline result relative to the Ręka et al. [21] cohort (*p* = 0.014) should be regarded as exploratory, whereas the differences from the larger phenotypically heterogeneous cohorts were more robust.

The absence of exome or genome sequencing data in individuals with negative CMA results precludes the identification of single-nucleotide variants and small indels that may contribute to the genetic etiology in a subset of patients, limiting a more comprehensive genomic characterization of the cohort.

In addition, the lack of parental segregation studies for the identified CNVs limits the interpretation of variants of uncertain significance (VUS) and CNVs with borderline clinical relevance. Inheritance status (de novo versus inherited) was not assessed in this cohort, although it represents an important line of evidence within ACMG guidelines that may contribute to variant reclassification. Family data have a further limitation. ASD among relatives was recorded at enrolment and is reported in Section 3.5, but the original study did not include a structured instrument for psychiatric disorders other than ASD in parents or other relatives; the K-SADS-PL was applied to the participants themselves, with a parent as informant. Psychotic, affective and other disorders in adult relatives, which bear on the multifactorial model of idiopathic ASD, were therefore not recorded in a form that permits systematic analysis. ASD in parents and in more distant relatives was also based on information given by the family rather than on standardized assessment, unlike the sibling diagnoses, which were confirmed within the study. Structured family psychiatric histories and parental segregation studies are planned for future work in this cohort.

Finally, the cohort included several sibling pairs, introducing a degree of non-independence among observations that was not accounted for in the descriptive and comparative analyses presented here. Not all siblings of enrolled probands underwent genetic testing, which further limits the assessment of variant segregation within families.

### 4.2. Clinical and Research Implications

The present findings have implications for both clinical practice and future genomic research in ASD. From a clinical perspective, the relatively low diagnostic yield observed in this carefully selected nonsyndromic cohort should not be interpreted as evidence against genetic testing. Even in individuals without dysmorphic features, major congenital anomalies, epilepsy, abnormal head circumference, or a clinically recognizable genetic syndrome, CMA identified recurrent CNVs with potential implications for genetic counseling, recurrence-risk assessment, and surveillance for associated neurodevelopmental, medical, and psychiatric manifestations. These findings support consideration of genomic evaluation beyond patients with overtly syndromic presentations, while emphasizing that pre-test counseling should address the expected variation in diagnostic yield according to phenotype.

The results also highlight the importance of interpreting CNVs within their broader clinical and familial context. Recurrent CNVs associated with ASD may show variable expressivity and incomplete penetrance, and some may function as susceptibility alleles rather than fully penetrant causal variants. Accordingly, parental testing and segregation analysis may provide important additional information for variant interpretation, recurrence-risk estimation, and genetic counseling, particularly for CNVs with reduced penetrance or uncertain clinical significance.

From a research perspective, future studies should combine detailed and standardized phenotypic characterization with family-based genomic analyses and sequencing approaches. The relatively low CMA yield in this clinically homogeneous cohort suggests that sequence-level variants not detectable by microarray may account for part of the unexplained genetic contribution to nonsyndromic ASD. Integration of CMA with exome or genome sequencing, together with systematic assessment of psychiatric phenotypes in participants and relatives, may help clarify the contribution of CNVs, sequence variants, and their interactions to the heterogeneous genetic architecture of ASD. Larger multicenter cohorts using harmonized phenotypic definitions will also be important to enable more meaningful comparisons of diagnostic yield across populations.

## 5. Conclusions

In this clinically well-characterized cohort of individuals with nonsyndromic ASD requiring Level 1 support, chromosomal microarray analysis (CMA) identified pathogenic or likely pathogenic CNVs in 2.8% of participants. Although the diagnostic yield was comparatively low, recurrent CNVs of clinical relevance were identified even in individuals without features suggestive of syndromic or complex neurodevelopmental presentations. These findings demonstrate that phenotypic selection substantially influences CMA diagnostic yield and should be carefully considered when interpreting and comparing results across ASD cohorts. Overall, this study provides one of the largest clinically well-characterized cohorts of individuals with nonsyndromic ASD evaluated by CMA in Brazil, contributing additional evidence on the diagnostic yield and potential clinical relevance of CMA in this specific population.

## Figures and Tables

**Table 1:** Demographic and diagnostic characteristics of the study cohort.

Characteristic	Value
Final CMA cohort, *n*	215
Age at study entry, mean ± SD	10.8 ± 3.0 years
Age at study entry, median (range)	10.3 (6.2–17.7) years
Sex, male, *n* (%)	166 (77.2%)
Sex, female, *n* (%)	49 (22.8%)
ASD diagnosis confirmed by ADI-R and ADOS-2, *n* (%)	215 (100%)
Pathogenic or likely pathogenic CNV, *n* (%)	6 (2.8%)
Variant of uncertain significance (VUS), *n* (%)	1 (0.5%)
Normal result (including incidental AOH), *n* (%)	208 (96.7%)

CMA: chromosomal microarray; ADI-R: Autism Diagnostic Interview–Revised; ADOS-2: Autism Diagnostic Observation Schedule, Second Edition; CNV: copy number variant; VUS: variant of uncertain significance; AOH: absence of heterozygosity. The male-to-female ratio (3.4:1) is in keeping with the approximately 4:1 ratio reported in the literature.

**Table 2:** Pathogenic and likely pathogenic copy number variants identified in participants with nonsyndromic autism spectrum disorder.

ID	Sex	Age at Assessment (y)	ASD and Psychiatric Phenotype	CNV	Genomic Coordinates (GRCh37/hg19)	Size	ACMG Classification
AUT168	Male	9	ASD	2q11.1–q11.2 deletion	chr2:96,766,560–98,013,866	1.24 Mb	Pathogenic
AUT26	Male	9	ASD, ADHD	2p16.3 microdeletion	chr2:51,158,241–51,491,417	333.2 Kb	Pathogenic
AUT80	Male	9	ASD, GAD, OCD; Tourette syndrome	15q11.2 deletion	chr15:22,698,522–23,300,287	601.8 Kb	Pathogenic
AUTi38	Male	16	ASD, ADHD; MDD, dysthymia, enuresis, ODD, persistent tic disorder	16p11.2 duplication	chr16:29,238,593–30,332,581	1.09 Mb	Pathogenic
AUT187	Male	9	ASD, ADHD. DMDD	6p22.3–p22.2 duplication	chr6:24,445,100–26,472,702	2.02 Mb	Likely pathogenic
AUT275	Female	11	ASD, ADHD	3q29 microdeletion	chr3:195,740,357–197,446,501	1.70 Mb	Pathogenic

CNV: copy number variant; ASD: autism spectrum disorder; ADHD: attention-deficit/hyperactivity disorder; OCD: obsessive–compulsive disorder; GAD: generalized anxiety disorder; MDD: major depressive disorder; DMDD: disruptive mood dysregulation disorder; ODD: oppositional defiant disorder. All participants met DSM-5 criteria for ASD requiring Level 1 support. Age refers to age at the time of standardized diagnostic assessment (ADI-R/ADOS-2), performed between 2019 and 2023. All CNVs were heterozygous. Genomic coordinates are based on the GRCh37/hg19 human reference genome.

**Table 3:** Psychiatric comorbidities identified using the K-SADS-PL among assessed participants (*n* = 210).

Comorbidity	*N*	%
ADHD	111	52.9%
Specific phobia	77	36.7%
Anxiety disorders	41	19.5%
Tic disorders/Tourette syndrome	35	16.7%
Depressive disorders/dysthymia	20	9.5%
Enuresis	16	7.6%
Encopresis	13	6.2%
ODD	8	3.8%

ADHD: attention-deficit/hyperactivity disorder; ODD: oppositional defiant disorder. Percentages are based on the 210 patients with an available CMA result and K-SADS-PL data; categories are not mutually exclusive, as patients could present more than one comorbidity.

**Table 4:** Clinical definitions, phenotypic selection criteria, sample sizes and chromosomal microarray diagnostic yields of the present cohort and of the reference cohorts used for comparison.

Cohort	Design and Recruitment Source	Clinical Definition and Phenotypic Selection Criteria	*n*	P/LP CNVs, *n* (%)	Comparison with Present Study
Present study (Curitiba, Paraná, Brazil)	Prospective recruitment from outpatient clinics; retrospective analysis of the collected data	Clinically nonsyndromic ASD confirmed by both the ADI-R and the ADOS-2. Excluded before CMA: dysmorphic features, known or suspected genetic syndrome, major congenital malformation, epilepsy, macrocephaly or microcephaly	215	6 (2.8%)	Reference
Tammimies et al. [20]—overall cohort	Population-based ASD cohort, Canada	All participants with ASD, unstratified, including dysmorphic and complex phenotypes	258	24 (9.3%)	OR 0.28; *p* = 0.004
Tammimies et al. [20]—“essential” subgroup	Same cohort, stratified by the Autism Dysmorphology Measure	Least dysmorphic stratum. Stratification was not exclusionary: 24.4% had macrocephaly and 11.7% had at least one major congenital anomaly	168	7 (4.2%)	OR 0.66; *p* = 0.57
Sandoval-Talamantes et al. [19]	Prospective cohort, tertiary hospital, Spain	“Primary” (nonsyndromic) ASD. 32% had intellectual disability or psychomotor delay and 6.6% had epilepsy	212	28 (13.2%)	OR 0.19; *p* < 0.001
Chaves et al. [11]—overall ASD cohort	Retrospective analysis of diagnostic CMA files processed in Florianópolis, Santa Catarina, between 2013 and 2019	ASD cited as the main reason for referral or as one phenotype of a broader spectrum, within 1012 individuals referred for neurodevelopmental disorders or congenital anomalies. ASD was not established using standardized instruments	333	35 (10.5%)	OR 0.24; *p* < 0.001
Chaves et al. [11]—“isolated ASD” subgroup	Same cohort, stratified post hoc	Nonsyndromic ASD without intellectual disability, defined by the absence of intellectual disability and of dysmorphic features or congenital anomalies, and restricted to individuals aged 5 years or over. Epilepsy, macrocephaly or microcephaly and recognized genetic syndromes were not exclusion criteria; and neither ASD nor intellectual disability was established using standardized instruments	145	7% (count not reported)	OR 0.40; *p* = 0.12
Ręka et al. [21]	Retrospective cohort, genetics and paediatric neurology clinics, Lublin, Poland	ASD by DSM-5 or ICD-10 criteria referred for CMA, with no phenotypic restriction: 4.3% had a recognized genetic syndrome	234	20 (8.55%)	OR 0.31; *p* = 0.014

ASD: autism spectrum disorder; CMA: chromosomal microarray analysis; P/LP: pathogenic or likely pathogenic; CNV: copy number variant; OR: odds ratio; ADI-R: Autism Diagnostic Interview–Revised; ADOS-2: Autism Diagnostic Observation Schedule, Second Edition. Odds ratios refer to the present cohort relative to each comparator. The *p* values come from two-sided Fisher’s exact tests and are nominal, without adjustment for multiple comparisons. The “essential” and “isolated ASD” subgroups were defined post hoc within their respective cohorts and used less restrictive selection criteria than the present study, and are therefore not equivalent to the clinically nonsyndromic cohort reported here. Chaves et al. [11] report a 7% yield for the isolated-ASD subgroup without giving the corresponding number of positive cases. The present cohort and that of Chaves et al. are independent samples rather than sequential analyses of the same participants: our participants were recruited prospectively from outpatient clinics in Curitiba and neighbouring municipalities in the state of Paraná, whereas Chaves et al. analysed CMA files processed between 2013 and 2019 by a diagnostic laboratory in Florianópolis, Santa Catarina. No participant is common to the two studies.

**Table 5:** Familial occurrence of autism spectrum disorder among the 215 participants.

Familial Occurrence of ASD	*n*	% of 215
Parent with ASD	9	4.2%
Mother	6	2.8%
Father	3	1.4%
Sibling with ASD confirmed in the study and tested by CMA (12 sibships)	24	11.2%
At least one sibling assessed in the study, irrespective of outcome	29	13.5%
At least one first-degree relative (parent or sibling) with ASD	31	14.4%
At least one relative of any degree with a reported ASD diagnosis	60	27.9%

ASD: autism spectrum disorder; CMA: chromosomal microarray analysis. Categories are not mutually exclusive, and first-degree relatives comprise parents and siblings. The sibling diagnoses in the fourth row were established within the study using the ADI-R and ADOS-2. ASD in parents and in more distant relatives was recorded from information provided by the family at enrolment and was not independently confirmed by standardized assessment. No structured instrument was applied to relatives for psychiatric disorders other than ASD.

## Data Availability

The data presented in this study are available on request from the corresponding author. The data are not publicly available due to privacy and ethical restrictions concerning identifiable genetic and clinical information of minors.

## Abbreviations

The following abbreviations are used in this manuscript:

ASD	Autism spectrum disorder
CMA	Chromosomal microarray analysis
CNV	Copy number variant
ADI-R	Autism Diagnostic Interview–Revised
ADOS-2	Autism Diagnostic Observation Schedule, Second Edition
ACMG	American College of Medical Genetics and Genomics
ADHD	Attention-deficit/hyperactivity disorder
AOH	Absence of heterozygosity
VUS	Variant of uncertain significance
WES	Whole-exome sequencing
SNP	Single nucleotide polymorphism
OCD	Obsessive–compulsive disorder
GAD	Generalized anxiety disorder
MDD	Major depressive disorder
DMDD	Disruptive mood dysregulation disorder
ODD	Oppositional defiant disorder
